# Spatial and Temporal Visualization of Polymorphic Transformations in Pharmaceutical Tablets

**DOI:** 10.1002/anie.202412976

**Published:** 2024-12-02

**Authors:** Julia Gasol‐Cardona, Martin R. Ward, Olof Gutowski, Jakub Drnec, Christian Jandl, Danny Stam, Andrew G. P. Maloney, Daniel Markl, Stephen W. T. Price, Iain D. H. Oswald

**Affiliations:** ^1^ Strathclyde Institute of Pharmacy and Biomedical Sciences University of Strathclyde 161 Cathedral Street Glasgow G4 0RE United Kingdom; ^2^ Centre for Continuous Manufacturing and Advanced Crystallisation (CMAC) University of Strathclyde 99 George Street Glasgow G1 1RD United Kingdom; ^3^ Deutsches Elektronen-Synchrotron DESY Notkestraße 85 22607 Hamburg Germany; ^4^ European Synchrotron Radiation Facility 71 Avenue des Martyrs 38000 Grenoble France; ^5^ ELDICO Scientific AG Switzerland Innovation Park Basel Area Hegenheimermattweg 167 A 4123 Allschwil Switzerland; ^6^ The Cambridge Crystallographic Data Centre 12 Union Road Cambridge CB2 1EZ United Kingdom; ^7^ Finden Limited Merchant House, 5 East St Helens Street Abingdon OX14 5EG United Kingdom

**Keywords:** X-Ray Diffraction Computed tomography, X-ray Diffraction, Pharmaceutical tablet, microcrystalline cellulose, phase transitions, pressure-induced polymorphism

## Abstract

X‐ray Diffraction Computed Tomography (XRD‐CT) represents a cutting‐edge method for non‐destructive material analysis, offering the unique capability of providing molecular‐level information with spatial resolution. In this study, we have applied XRD‐CT to investigate pharmaceutically relevant tablets that have been subjected to a range of compression pressures typical in tablet manufacturing. By employing XRD‐CT to pharmaceutical tablets, we reveal material changes without tablet destruction, thereby avoiding potential phase transformations during sample preparation that could lead to errors in the interpretation of the processes that have occurred. Utilizing a pressure‐sensitive marker, glycolide, we have tracked changes within tablet structures induced by compression, pinpointing locations where glycolide undergoes pressure‐induced transformation. Additionally, we conducted a follow‐up study with analysis one month later, observing an in situ hydrolysis reaction of glycolide within the tablets. Through the complementary use of electron diffraction, we have elucidated the structure of the hydrolysis product, further enhancing our understanding of temporal changes in the tablets.

## Graphical abstract

X‐ray Diffraction Computed Tomography (XRD‐CT) has been used to reveal pressure‐induced phase transformations in pharmaceutical tablets of glycolide as a function of compaction pressure. Temporal analysis of the tablets, and subsequent identification using electron diffraction, revealed a hydrolysis reaction occurred on storage of the tablets. This reaction was enhanced in the presence of microcrystalline cellulose.

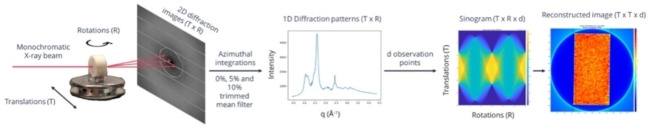

## Introduction

Compression‐induced phase transformations in pharmaceutically‐relevant organic materials is an area of current interest due to the increasing number of compounds that undergo such changes in pressure ranges relevant to pharmaceutical manufacturing processes.[^1^, ^2^, ^3^, ^4^] These changes have a material impact on the functionality of the compounds giving rise to potential changes in, for example, mechanical properties^[5]^ and solubility.^[6]^ Changes to the solid‐form have also been observed at pressures relevant to tableting, including polymorphic transformations (e.g. chlorpropamide[^7^, ^8^]), partial amorphisation (e.g. theophylline, nitrofurantoin, amlodipine besylate^[9]^), and crystallization of amorphous drugs (e.g. indomethacin, sucrose, celecoxib[^1^, ^10^]), using a range of characterization techniques including differential scanning calorimetry (DSC), spectroscopy and X‐ray and neutron diffraction. Investigations of pure substances have shown that the thermodynamic stability of polymorphs can change as a function of pressure^[11]^ but, in addition, the type of pressure (isotropic vs anisotropic) is important and can promote or inhibit transformation e.g. ammonium nitrate.[^12^, ^13^] These changes have been observed not only for pure components but also within formulated tablets composed of drug‐excipient mixtures to create robust compacts that mimic oral solid dosage forms for delivery to patients.

In the context of tableting, phase transformations are induced through the exertion of an axial pressure on the powder which causes both normal and shear stresses. The shear stress is imparted by the friction between tablet surfaces and die wall and punches, which results in density gradients of the final tablet with denser regions closer to the surface.^[8]^ Variables such as excipients, lubrication method, and compression conditions, e.g. pressure and dwell time, can all be explored to mitigate changes to the API solid form during this process.^[14]^ Temperature can also play a role with Infrared studies showing an increase of up to 10 °C post ejection.[^15^, ^16^, ^17^, ^18^, ^19^] However, once a tablet is formed there are limited methods by which these tablets can be analyzed at the crystal structure level. Phase transitions during compaction have required the break‐up of the tablet and/or trituration of the powders and subsequent X‐ray powder diffraction analysis to be able to observe the changes in the samples. Thakral et al.^[8]^ were able to reduce the sample preparation by cutting open the tablet and conducting 2D X‐ray diffraction to map the changes across the tablet and spatially resolve polymorphic forms. This method still requires the need for sample preparation where changes to the product can occur leading to an uncertainty over the impact of the compaction process versus the subsequent treatment of the sample. X‐ray Computed Tomography (X‐ray‐CT) is a method that has been used as a non‐destructive analytical tool to investigate a wide range of research questions associated with pharmaceutical dosage forms. Using the variation in density, pressure gradient,[^14^, ^20^] coatings,^[21]^ brittleness of components^[22]^ and agglomerates from crystallization,^[23]^ the components of a dosage form can be spatially resolved. Interesting work evaluating the particle shape and how that can be utilized in X‐ray‐CT to spatially resolve polymorphs in a sample has been conducted and shown to 99 % accuracy for clopidogrel bisulphate in a Plasdone S630 (N‐vinyl‐2‐pyrrolidone and vinyl acetate) capsule.^[24]^ Several studies have explored the use of X‐ray‐CT to evaluate the impact of typical processes such as compaction^[25]^ and dissolution.[^26^, ^27^, ^28^, ^29^] Specific to this paper, Fu et al. used glass beads as tracers to evaluate the way in which a powder was compressed in a tablet die with force applied from a single direction. Using X‐ray‐CT the authors were able to follow the trajectory of glass tracer particles due to the difference in density with the sugar spheres that made the bulk of the tablet and enabled the visualization of the compression pattern. This study revealed that the distribution of compaction force was not uniform and that there was significant movement of powder on the side of the movable piston and interestingly virtually no movement around the base.^[25]^ While these detailed studies have demonstrated that X‐ray‐CT is a powerful tool for visualization, they require a contrast in the density of the solids or particle morphology to be able to resolve the solid mixtures, which is an issue in organic molecules where the density contrast between polymorphs, or even amorphous content, is very small.[^30^, ^31^] Critical to the resolution of this issue is crystallographic information. X‐ray Diffraction Computed Tomography (XRD‐CT) can provide this chemical and spatially resolved information, enabling the distribution of different components in a sample to be readily identified.

XRD‐CT has been successfully applied to a number of material science areas to elucidate the composition of batteries,^[32]^ catalysts,^[33]^ energy storage materials, and even biomineralization in human bone structures.^[34]^ These studies have not only provided details under ambient conditions but also during working processes.^[33]^ These studies provide spatially‐resolved physico‐chemical information from within the interiors of intact objects negating the need for any intrusive sample preparation. In contrast to conventional absorption contrast tomography which uses a full‐field approach to provide information on density/absorption differences within a sample, XRD‐CT uses a pencil‐beam scanning approach to measure the average diffraction signal through the sample at a given point. With the rotation of the sample and reconstruction of the images from the data, a full crystallographic description of the sample that is spatially resolved can be provided.^[33]^

**Figure 1:**
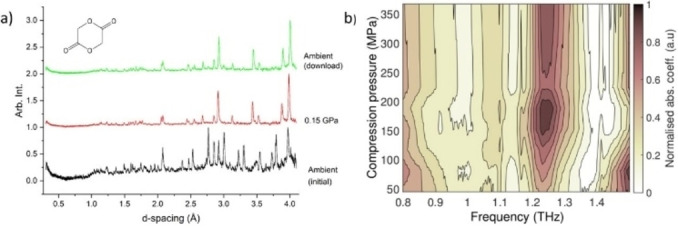
(a) Neutron powder diffraction patterns of glycolide at ambient and 150 MPa and its recovery to ambient pressure. (Reproduced with permission of the International Union of Crystallography).^[36]^ (b) Terahertz time‐domain spectroscopy (THz‐TDS) of a tablet of glycolide. A change in absorption coefficient at 150 MPa indicates the phase transition has occurred.

A recent review of pharmaceutical tablets using X‐ray tomography highlighted the lack of investigations using XRD‐CT into these systems.^[31]^ There is a significant challenge in this area due to the general low symmetry, large unit cell size, and poorer crystallinity often observed for softer organic materials. Here, we address this challenge and have applied XRD‐CT to pharmaceutical tablets, which have been a significant omission as the field has developed. In particular, we explore the evolution of polymorphic forms in pharmaceutical tablets as a function of compaction pressure using glycolide as a pressure‐sensitive marker. Glycolide undergoes a reconstructive phase transition at 150 MPa to a high‐pressure polymorph (Form II) that is recoverable to ambient conditions which makes it an ideal pressure marker for our ex situ measurements (Figure 1a).[^35^, ^36^] The pressure of this phase transformation is typical for an operating pressure in a tableting press used in the pharmaceutical industry. It should be noted that glycolide Form II has since been isolated at ambient pressure from cooling of a 2‐propanol solution from 75 °C to 35 °C and is sufficiently stable to perform solubility measurements.[^37^, ^38^]

Using XRD‐CT we have been able to visualize the impact of compaction pressure on the contents of the tablet. These data indicate that the changes are far from uniform across the tablet. Serendipitously, we have been able to observe the chemical degradation and subsequent crystallization of the hydrolytic ring opening product of glycolide, 2‐(2‐hydroxyacetoxy)acetic acid, in situ within a tablet on storage which has not been observed to date.

## Results and Discussion

The initial structural investigations[^35^, ^36^] of glycolide were performed on the pure material itself using a liquid pressure‐transmitting medium to ensure that the structural information could be extracted under hydrostatic or pseudo‐hydrostatic conditions. These conditions are not typical in a tableting environment, hence we needed to demonstrate that the transition would still occur in the presence of excipients and under conditions of tableting. An initial test was performed on an in‐house compaction simulator (HB50, Huxley Bertram Engineering Ltd) and using a terahertz time‐domain spectroscopy (THz‐TDS) system (TeraPulse Lx Spectrometer, TeraView Ltd). A formulation containing glycolide (20 % wt.), microcrystalline cellulose (VIVAPUR® PH‐112; 79 % wt.) and magnesium stearate (1 % wt.) was loaded into the compaction simulator before applying incremental load and characterizing tablets offline using the THz‐TDS system. The resulting spectra enable the measurement of the absorption coefficient of the solids on increasing pressure hence it can be inferred that an observable discontinuous change can be attributed to a phase transformation in the material of study.^[39]^ There is a clear discontinuous change to the spectra on compression above 150 MPa even in the presence of the excipients (Figure 1b) warranting further investigation using XRD‐CT.

To evaluate the effect of compaction pressure and obtain structural information on the degree of polymorphic transformation in glycolide tablets, we compressed 47 tablets to pressures ranging from 47 to 548 MPa with 20 tablets used for analysis. Blended powders were weighed and manually loaded into the die to compress the tablets to target pressures of 50, 100, 130, 150, 170, 200, 300, 400 and 548 MPa (see section S1.4 of Supporting Information). The weight of the tablet was altered to ensure that the tablet thickness was constant while varying the compaction pressure. Flat‐faced round steel punches (diameter: 5 mm) were used to compress the powder over a 1 s period using a uniaxial compression profile. The tablets were prepared 4 days in advance of characterization on beamline P07‐DESY at PETRA III.^[40]^ Between preparation and characterization, the tablets were stored in a sealed container at 5 °C±2 °C and 65 %±5% relative humidity. The tablets underwent full relaxation before being studied.^[41]^

**Figure 2:**
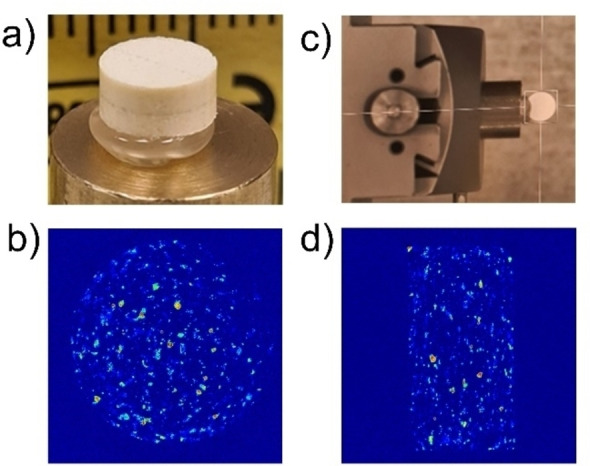
(a) Example tablet (not glycolide tablet) mounted on the goniometer at P07‐DESY at PETRA III. The dark lines on the tablet indicate the beam path during experiment; (b) XRD‐CT image of a tablet as orientation in a). The colour scheme indicates the presence of the Form I in the tablet (blue‐red from absence to presence). (c) second orientation of the tablet indicating where the tablet was glued to the mount; (d) image of the tablet using colour map of presence of Form I; same colour scheme to (b)).

The tablets were mounted onto a standard Huber goniometer in two orientations to provide lateral and longitudinal cross sections (Figure 2a, c) using the XRD‐CT method which typically takes ca. 1 hour for the data collection; full experimental details are included in the SI. Phase identification of the reconstructed XRD‐CT datasets was performed on summed global patterns using X'Pert HighScore Plus,^[42]^ with the phases identified as being glycolide Form I (CSD refcode NAHNIT01), glycolide Form II (CSD refcode NAHNIT02), microcrystalline cellulose and an unknown phase, later identified to be 2‐(2‐hydroxyacetoxy)acetic acid. Magnesium stearate was added at a low level of 1 % wt. in the powder blend so its presence in the tablets was sparsely detected. Unique reflections were identified and used to generate the spatial distribution maps of each phase.

**Figure 3:**
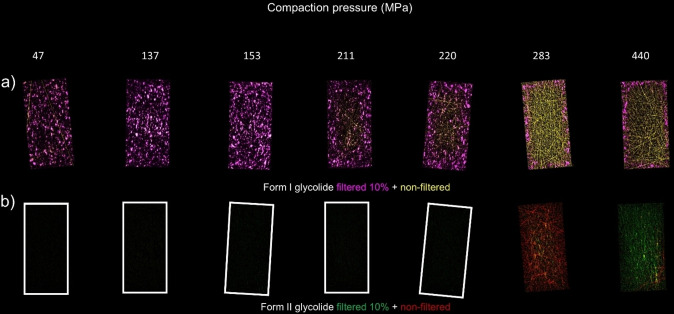
XRD‐CT images of tablets compacted at different pressures. (a) Form I glycolide filtered 10 % (pink) and non‐filtered (yellow). (b) Form II glycolide filtered 10 % (green) and non‐filtered (red). The images for pressure points 47–220 have been given borders to outline the edge of the tablets due to the scale of the data.

A selection of tablets across the 47 to 548 MPa range of compaction pressures were subject to analysis and their composition maps created. We have limited the discussion to the side profile of the tablet, shown in Figure 2d, however, similar observations are found for the front‐facing direction. The reconstructed images of the tablets (Figure 2d) clearly show the spatial distribution of the phases, where normalised glycolide Form I distribution is represented by a jet colour Scheme. The diffraction patterns indicate that, of the known forms of cellulose, the MCC present can be identified as Cellulose I.^[43]^ Cellulose I can be split into two crystallographically independent phases, I_α_ and I_β_;[^44^, ^45^, ^46^] polymorph I_β_ is the more thermodynamically stable form.^[47]^ Figure 3 shows the XRD‐CT images showing distribution of Form I and II for tablets compressed at different pressures. If we focus on the low pressure image of, 47 MPa, it shows that the components of the tablet are homogeneously mixed together with an even distribution of the Glycolide Form I in the bulk MCC. MCC appears to remain unaffected and its distribution is consistently maintained across all pressures (see Supporting Information for maps of MCC) which supports previous observations[^48^, ^49^] hence the discussion will focus on the transformations in glycolide (Figure 3a).

A key consideration in XRD‐CT is the way in which data are treated. XRD‐CT requires good, well‐distributed powders so that the reconstruction is not impacted by diffraction events from single crystals. These events manifest themselves in “streak” artefacts in the reconstructed images and distort them, hence we need an approach to counter this without compromising the information in the patterns.^[50]^ Organic materials, including glycolide, can be poor powders with larger particles present despite significant particle size reduction through milling. Typically, this problem is addressed via a mean‐trimmed filtering approach which removes values above/below a specified limit, e.g. the top and bottom 10 % of diffraction intensity at any given 2Θ value. By this approach, the significant individual spots are removed whilst, crucially, maintaining the diffraction from the rest of the powder at the same 2Θ range. This process leads to significantly improved image quality in the reconstructions.

Consideration of the filtered data alone provides clear distributions in the reconstructed images (Form I, pink; Form II, green; Figure 3). As the compaction pressure is increased, we begin to see a change to the intensity and distribution of Form I (pink, Figure 3a). The observed intensities of Form I diminish in the center of tablets compressed to higher pressures but are present at the edges of the tablet. This change clearly begins between 153 and 211 MPa. At the same time, the contribution from Form II is not significant until 283 MPa (green). The pressure at which the changes occur is commensurate with the previous diffraction studies but there is a difference between the pressures at which these events occur indicating that they cannot be correlated. To help explain this we need to consider the non‐filtered data as this reveals some significant findings to enhance our understating of the changes.

The non‐filtered data (yellow, Form I; red Form II) reveals an increase in the presence of larger crystallites in the center of the tablet, those for Form I start to appear at 211 MPa reaching a maximum at 283 MPa before diminishing at 440 MPa. This indicates that at higher compaction pressures the original powder particles are annealing together to form larger particles when subjected to compression. Glycolide is a van der Waals solid which may facilitate this process due to the weaker, more malleable intermolecular interactions. This phenomenon is similar to the behavior observed in other van der Waals solids like benzene when solidified under pressure.[^51^, ^52^] Particle agglomeration, dominated by solid bridge formation, has also been reported during the compression of micronized small‐molecule APIs like furosemide and griseofulvin blended with excipients.^[53]^ Although the author did not discuss the single‐crystal‐like nature of the agglomerates, Potharaju noted that the agglomerate size increased steadily with increasing compression force, paralleling the observed trend for glycolide Form I.

The question remains, is the transition to Form II observable in tablets compressed to pressures in excess of 150 MPa as was proposed? From the data, we observed a stark change to the diffraction attributable to Form II at 283 MPa and beyond. At 283 MPa, there is an increase in the non‐filtered powder diffraction for Form II (red, Figure 3b) which may be accounted for by the transformation of the larger annealed crystals from Form I to Form II. The compaction pressure at which we observe the changes correlates reasonably well with those from our initial neutron diffraction and terahertz spectroscopy experiments.^[36]^ At higher tableting pressures (440 MPa) we observe less streaking for both Form I and II suggesting that the particles undergo fragmentation into smaller particles providing better powder diffraction. There is clear evidence of a greater proportion of Form II across the tablet at this higher compaction pressure (more intense color Figure 3b).

From our observations, there is approximately a 50 MPa difference between the point where a change in the distribution of Form I is evident, and the phase transition pressure of glycolide under hydrostatic conditions. For a pure glycolide compact, we would anticipate a lower phase transition pressure upon compaction, given the reduced size of glycolide particles^[36]^ and the increase in shear stress upon anisotropic compression. Thakral et al. observed that when chlorpropamide in a blend with a significant MCC content (50 : 50 wt %), the presence of MCC reduced the extent of transformation between polymorphs.^[8]^ Though the reduction observed in the Thakral study was subtle, we report a more pronounced impact of the formulation on transformation pressure as it contains a higher MCC content (80 % wt). Li et al. have also shown that magnesium stearate presence in blends can also alter the compaction pressure required to induce a phase transformation.[^8^, ^54^]

One of the key observations of this study is that the retention of Form II on decompression has enabled us to observe the impact of pressure on the system without the destruction of the tablet. Form II is still the thermodynamically less stable form under ambient conditions, hence there is a driving force to transform back to Form I. The local environment, i.e. whether it is in the center or the edge of the tablet, will play a role in the kinetics of the transition and we observed this in the image of Form I around the edge at 211 MPa. Those crystallites at the edges of the tablet are much more likely to convert to Form I due to the interaction with moisture in the air and also through shear force interaction with the die holder of the tablet press. Both of these interactions can facilitate the transition back to the ambient form.[^55^, ^56^] The Form I to Form II transition is reconstructive where bigger particles are broken up into smaller particles,^[57]^ even if micron sized. A result of this is that the powder produced should become a “better” powder for X‐ray diffraction by reducing any preferred orientation that may be present initially. Both of these factors aid the analysis using XRD‐CT as there is less requirement to filter out single crystal diffraction providing a more optimal reconstruction of the image (for details see the SI). We observe this through a reduced number of single crystal particles around the edges of the tablet and the increased resolution of the border around the edge of the tablet as increasing compaction pressure is used.

Due to the allocation of synchrotron beamtime, we had the opportunity to explore the effect of storage time on the same tablets to observe whether the conversion back to Form I would occur. Stability testing of pharmaceutical products is a critical component of the drug development process. As part of this study, we have been able to follow drug‐excipient compatibility^[58]^ as a function of time by collecting data one month after the initial compaction. Where possible, we used the same tablets that were used initially but, in some cases, this was not possible due to the tablet fracturing while unmounting the sample from the goniometer. The tablets were stored under the same low temperature and high relative humidity conditions. Overall, visually, the samples showed a significant increase in the roughness of the overall tablet surface upon 1 month of storage.

**Figure 4:**
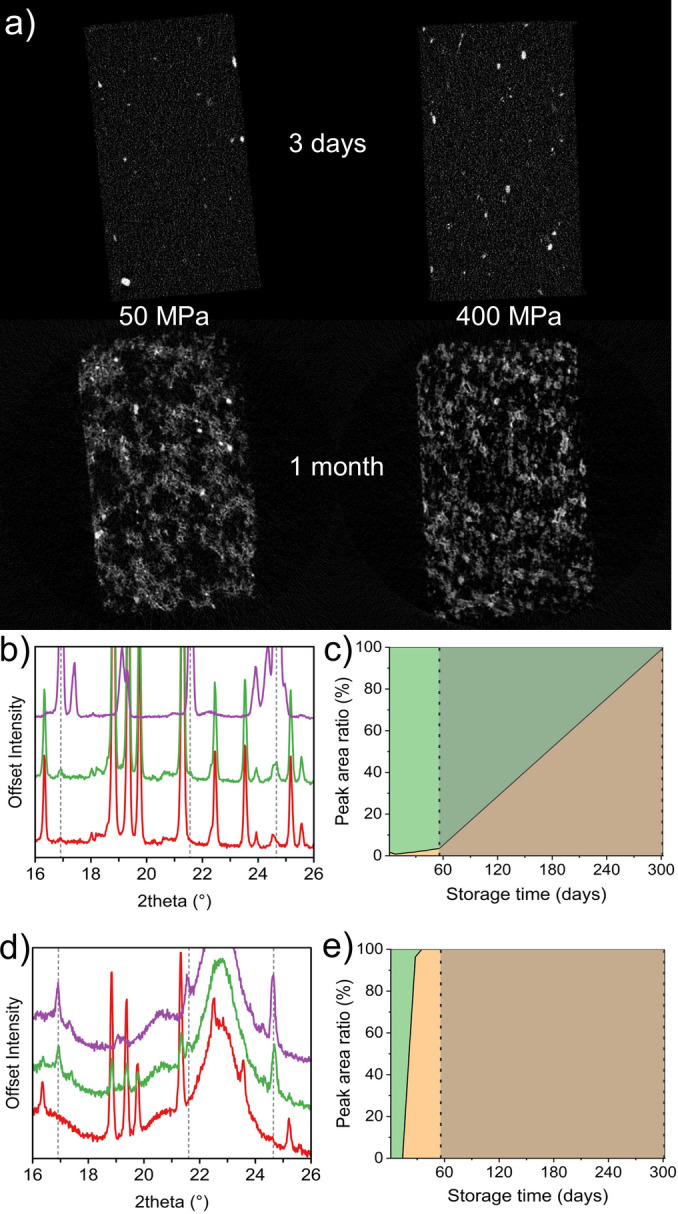
(a) X‐ray Diffraction CT images for the tablets compressed to 50 MPa and 400 MPa at t=4 days (Fresh) and t=30 days (aged).. The white particles indicate the presence of 2‐(2‐hydroxyacetoxy)acetic acid. The XRPD patterns of the pure (b) and blended (d) mixtures are shown for day one (red), three weeks (green) and 10 months (purple) of storage. The change in the area under two characteristic peaks in the XRPD patterns of glycolide Form I ([002]; green area) and 2‐(2‐hydroxyacetoxy)acetic acid ([100]; orange area) is tracked in the pure (c) and blended (e) mixtures over time and compared as a ratio.

XRD‐CT of all the samples measured indicates a significant change to the structure of the components of the tablets (Figure 4a). Firstly, both forms of crystalline glycolide deteriorated over the course of a month as there was no indication of either Form in the tablet. Secondly, the peak height and sharpness of the diffraction pattern of an unknown phase were both enhanced in the aged samples indicating increased formation of this crystalline solid during storage, as seen in Figure 4a for two different compaction pressures. Interestingly, additional diffraction patterns isolated from parts of the tablet cannot be identified as any of the known forms of any of the components. In these images, the white areas represent the distribution of the unknown phase; a comparison of the global diffraction pattern with the respective phase is shown in Figure S5. It can clearly be observed that there is a small quantity of this phase in the fresh tablets, but the aging has caused a significant increase throughout the tablet. It is reasonable to assume from the extent of observation that it is related to either the glycolide or the MCC content based on the initial weights in the tablet. Considering MMC initially, the diffraction pattern shows that Cellulose I[^44^, ^45^, ^59^, ^60^] is present as a mixture of the two different polymorphs, I_α_ and I_β_.[^44^, ^45^, ^47^] The unknown phase has a sharper diffraction pattern from a more crystalline product indicating conversion to either one of the two polymorphs. Cellulose I_β_ is the more likely choice due its higher stability (approx. 8 kJ/mol[^46^, ^47^, ^61^]) and the pair of Bragg reflection above 2Θ=16° and those above 2Θ=20° are somewhat more discriminatory (Figure S5). Conversion of MCC during storage has not been observed during stability studies[^49^, ^62^, ^63^] and the only transition reported from I_α_ to I_β_ was after an annealing process at 260 °C using NMR as a probe^[64]^ hence it is unlikely that the new material has its origin from MCC.

The disappearance of the two forms of glycolide suggests that the unknown product is related to this either as a new solid‐state form of glycolide e.g. polymorph, hydrate etc. or a reaction product. Separate time‐dependent stability studies of glycolide powder were performed in‐house. Glycolide was stored as the pure phase and as the blended mixture under the same conditions as the tablets used in the study (5 °C and 65 % RH). The powder diffraction study (Figure 4b‐e) shows that glycolide converts to the same unknown phase over the course of a few weeks. In both the pure and blended mixtures, the conversion to the unknown phase begins after two to three weeks but the extent of conversion appears to be accelerated in the blended product with complete conversion after four weeks where the conversion of the pure phase is complete after 10 months. No data were collected between 2 and 10 months due to assumed stability but 3.6 % conversion had occurred at 2 months in the pure sample. The conversion of the pure phase enabled indexing of the sample with unit cell parameters of *a*=5.28(5) Å, *b*=5.35(5) Å, *c*=19.92(19) Å, β=90.900(13)°, volume=562(9) Å^3^ and space group *P*2_1_/*c*. Unfortunately, the structure could not be solved from the powder diffraction data but the crystal quality was good enough to use the recent developments in electron diffraction to solve the crystal structure of the unknown phase.[^65^, ^66^] Full experimental details are included in section S1.6 of the Supporting Information.

**Figure 5:**
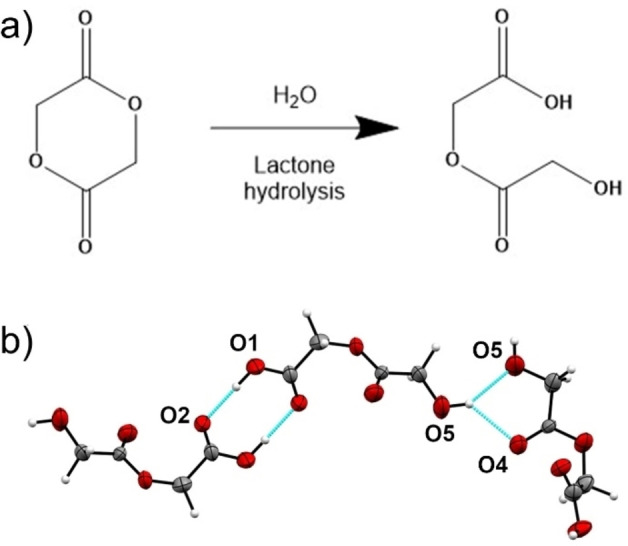
Lactone hydrolysis of glycolide to 2‐(2‐hydroxyacetoxy)acetic acid (a), and Hydrogen bond motifs in the crystal structure of 2‐(2‐hydroxyacetoxy)acetic acid (b).

The unknown phase from our stability studies is 2‐(2‐hydroxyacetoxy)acetic acid (HAA), the hydrolytic ring opening product of glycolide (Figure 5). The crystal structure from the ED data provides an excellent fit to the XRPD patterns collected as part of the stability study and as tablet, confirming phase purity (Figure S10). HAA crystallizes in monoclinic space group *P*2_1_/*c* with one molecule in the asymmetric unit. HAA molecules hydrogen‐bond through O1‐H…O2 H‐bonded dimers with an R^2^_2_(8) graph set notation^[67]^ and O5‐H…O4/O5 bifurcated H‐bond, forming a layered herringbone structure. To the best of our knowledge, this is the first time this structure has been reported.

Having determined the source of the new diffraction data we can begin to understand the process of the conversion between glycolide and HAA. We postulate that the hygroscopic nature of MCC increases the moisture content inside the blend to drive and increase the rate of hydrolysis. Suzuki and Nakagami^[62]^ showed that MCC with a higher amorphous content was more hygroscopic than with a higher crystalline content (25 % vs. 65 % crystallinity) with an ~8 % increase in moisture uptake for the higher amorphous content MCC. In general, cellulose has a crystallinity that varies between 53–82 %[^68^, ^69^] depending on the source of the MCC. In addition, glycolide is hygroscopic itself which will enhance the uptake of moisture hence conversion was observed in the pure phase as well, albeit more slowly. The stored tablets appeared to have a rougher surface compared to their initial state which can be accounted for by the recrystallization of HAA as the hydrolysis reaction occurred. The low temperature storage conditions and slower rate of crystal growth will have contributed to the production of a highly crystalline powder. Over this time the presence of MCC within the tablets does not change hence the degradation of the glycolide does not appear to have affected the MCC. Our observation is supported by previous investigations into various grades of MCC using X‐ray diffraction, which did not show any significant change to the diffraction pattern despite being subject to various environments.^[49]^

## Conclusion

Our study showcases the effective utilization of XRD‐CT methodology in spatially resolving components with similar densities within pharmaceutical tablets, offering a detailed map of their composition. Despite the challenges posed by deciphering diffraction patterns of low symmetry systems and low molecular weight, we have successfully observed phase transitions directly in these tablets without resorting to sample manipulation such as trituration of tablet into powder, thus avoiding potential polymorphic transitions in the compounds. By employing a pressure‐sensitive compound, we have pinpointed the precise locations of phase transitions and assessed the impact of compaction pressure on formulation. Notably, our approach has enabled both temporal and spatial resolution of changes occurring in these tablets. Furthermore, our investigation serendipitously uncovered a solid‐state chemical reaction within the tablet, wherein the hydrolytic ring opening product of glycolide, 2‐(2‐hydroxyacetoxy)acetic acid (HAA), was first identified using XRD‐CT and subsequently confirmed through electron diffraction, culminating in the elucidation of its crystal structure. In the wider context of pharmaceutical research, our ability to apply this technique to pharmaceutically relevant formulations will enable a deeper, more intimate understanding of the effect of tableting pressure on the formulation in pharmaceutical products. The molecular level of insight, without the destruction of the sample, is a step change in the way in which we are able to view formulated products.

## Supporting Information

The authors have cited additional references within the Supporting Information. Deposition Number 2364365 contains the supplementary crystallographic data for this paper. These data are provided free of charge by the joint Cambridge Crystallographic Data Centre and Fachinformationszentrum Karlsruhe Access Structures service and can be accessed at www.ccdc.cam.ac.uk/structures.

## Conflict of Interests

The authors declare no conflict of interest.

1

## Supporting information

As a service to our authors and readers, this journal provides supporting information supplied by the authors. Such materials are peer reviewed and may be re‐organized for online delivery, but are not copy‐edited or typeset. Technical support issues arising from supporting information (other than missing files) should be addressed to the authors.

Supporting Information

Supporting Information

## Data Availability

Parts of this research were carried out at beamline P07‐DESY at PETRA III. Beamtime was allocated for proposal I‐20220327. No Data DOI is available for these datasets. We acknowledge the European Synchrotron Radiation Facility (ESRF) for provision of synchrotron radiation facilities under proposal number CH6398 on ID31 (10.15151/ESRF‐ES‐902111435). Lab data underpinning this publication are openly available from the University of Strathclyde KnowledgeBase at https://doi.org/10.15129/802db8b4‐4593‐4e26‐a368‐021d70201d3e
