# Supplementary material for: Spatial and Temporal Visualization of Polymorphic Transformations in Pharmaceutical Tablets
Source: Angew Chem Int Ed Engl. 2024 Dec 2;64(2):e202412976. doi: 10.1002/anie.202412976 (PMC11720378; doi:10.1002/anie.202412976)
Supplement: Supplementary file 2 — Supporting Information [file ANIE-64-e202412976-s002.pdf]

Angewandte Chemie
Eine Zeitschrift der Gesellschaft Deutscher Chemiker

Supporting Information

Spatial and Temporal Visualization of Polymorphic Transformations in Pharmaceutical Tablets

J. Gasol-Cardona, M. R. Ward, O. Gutowski, J. Drnec, C. Jandl, D. Stam, A. G. P. Maloney, D. Markl, S. W. T. Price, I. D. H. Oswald**

Supplementary Information

Spatial and Temporal Visualization of Polymorphic Transformations in Pharmaceutical Tablets

Julia Gasol-Cardona,[a][b] Martin R. Ward,[a][b] Olof Gutowski,[c] Jakub Drnec,[d] Christian Jandl,[e] Danny Stam,[e] Andrew G.P. Maloney,[f] Daniel Markl,[a][b] Stephen W.T. Price[g]* & Iain D.H. Oswald[a]*

[a] Miss J. Gasol-Cardona, Dr M.R. Ward, Prof D. Markl & Dr, I.D.H Oswald
Strathclyde Institute of Pharmacy and Biomedical Sciences,
University of Strathclyde,
161 Cathedral Street,
Glasgow,
G4 0RE,
United Kingdom
E-mail: iain.oswald@strath.ac.uk

[b] Miss J. Gasol-Cardona, Dr M.R. Ward & Prof D. Markl
Centre for Continuous Manufacturing and Advanced Crystallisation (CMAC),
University of Strathclyde,
Glasgow
G1 1RD,
United Kingdom

[c] Mr O. Gutowski
Deutsches Elektronen-Synchrotron DESY,
Notkestraße 85,
22607 Hamburg,
Germany

[d] Dr J. Drnec
European Synchrotron Radiation Facility,
71 Avenue des Martyrs
38000 Grenoble,
France

[e] Dr C. Jandl & Dr D. Stam
ELDICO Scientific AG,
Switzerland Innovation Park Basel Area,
Hegenheimermattweg 167A,
4123 Allschwil,
Switzerland

[f] Dr A.G.P. Maloney
The Cambridge Crystallographic Data Centre,
Cambridge,
CB2 1EZ,
United Kingdom

[g] Dr S.W.T. Price
Finden Limited
Merchant House,
5 East St Helens Street,
Abingdon,
OX14 5EG,
United Kingdom
E-mail: stephen@finden.co.uk

Table of Contents

S1. Materials and Methods 3
S1.1 Materials 3
S1.2 Particle size reduction 3
S1.3 Powder characterization 3
S1.4 Tablet formulation 4
S1.4.1 Tablet Preparation 4
S1.4.2 Tablet storage 6
S1.5 X-ray Diffraction Computed Tomography 6
S1.6 Interpretation of the unknown phase 8
References: 15

S1. Materials and Methods

S1.1 Materials

Glycolide (1,4-dioxane-2,5-dione, 97%, CAS no. 502-97-6) was purchased from Thermo Fisher Scientific UK as a polycrystalline powder. VIVAPUR® 112 microcrystalline cellulose (MCC), purchased from JRS Pharma (CAS no. 9004-34-6), was used as filler. VIVAPUR® PH-112 is a commercial grade of MCC not typically used in direct compression formulations. However, its low moisture content helps reduce the occurrence of water-sensitive changes in the formulation. Technical grade magnesium stearate (MgSt), purchased from Sigma Aldrich UK (CAS no. 557-04-0), was added as an internal lubricant to prevent sticking during compression. Both excipients were used without further treatment. All the materials used in this formulation are hygroscopic.

S1.2 Particle size reduction

Glycolide was cryomilled using a Retsch CryoMill with a 25 mL stainless steel grinding jar and a single 15 mm diameter stainless steel grinding ball. Glycolide (2.00 g) was pre-cooled and milled at -196 °C (i.e., under a constant flow of liquid nitrogen from a 50 L Dewar). The material was pre-cooled for 5 min. at 5 Hz, then milled for 9 cycles of 5 min. at 25 Hz, with each milling cycle alternated with a 5 min. cycle at 5 Hz to prevent overheating of the sample. Once micronized and before blending, the material obtained was stored in an airtight container at 5 °C to prevent chemical degradation.

S1.3 Powder characterization

The phase purity of glycolide (both pre- and post-micronization), MCC and MgSt was checked and confirmed in-house using powder X-ray diffraction (XRPD). Samples of glycolide were not ground prior to analysis to prevent triggering the phase transition. XRPD data of a small quantity of sample (10 – 50 mg) was collected at ambient temperature on a Bruker D8 Discover diffractometer, configured in a Deybe-Scherrer transmission geometry and equipped with a SSD160-2 1D detector and an automated multiposition x-y sample stage. The sample was placed on a 40 position well plate lined with a 7.5 µm Kapton film and was scanned across a 4 – 40° 2θ range with a 0.017° 2θ step size and a 0.5 s per step count time. Samples were oscillated in the x-y plane at a speed of 0.03 mm/s throughout data collection to maximize powder sampling and minimize preferred orientation effects. X-ray emission was supplied by a Cu source (λ = 1.54060 Å, sealed tube operated at 40 kV and 10 mA) paired with a $K\alpha_{1,2}$ primary focusing Göebel mirror monochromator.

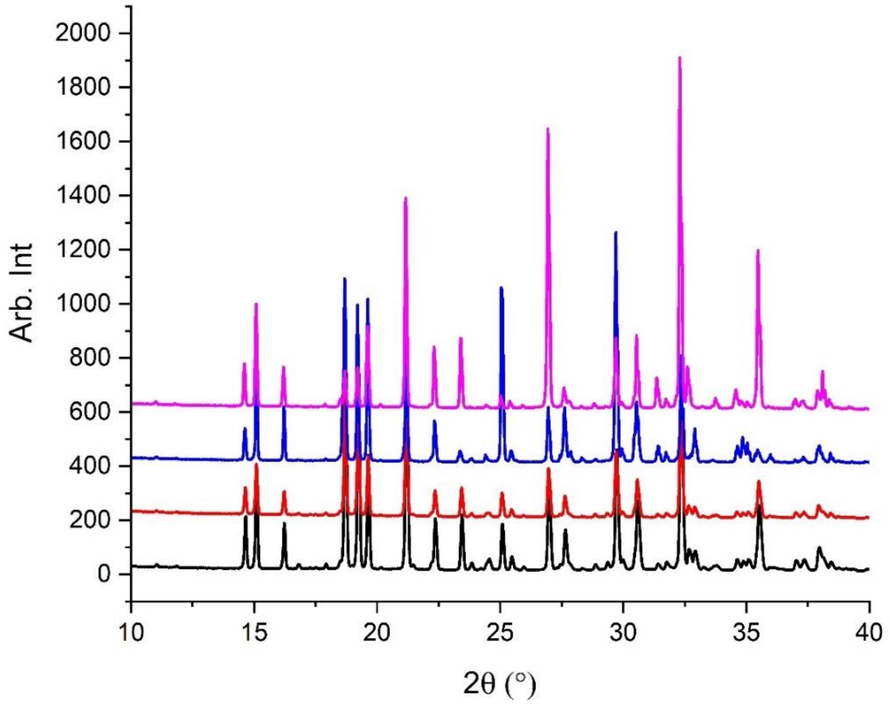

Figure S1 X-ray diffraction data for glycolide from the original source with (black and red) and without (blue and purple) cryomilling. Cryomilling has induced some conversion to the hydrolysis product (2-(2-hydroxyacetoxy)acetic acid; HAA) (peak at 17°).

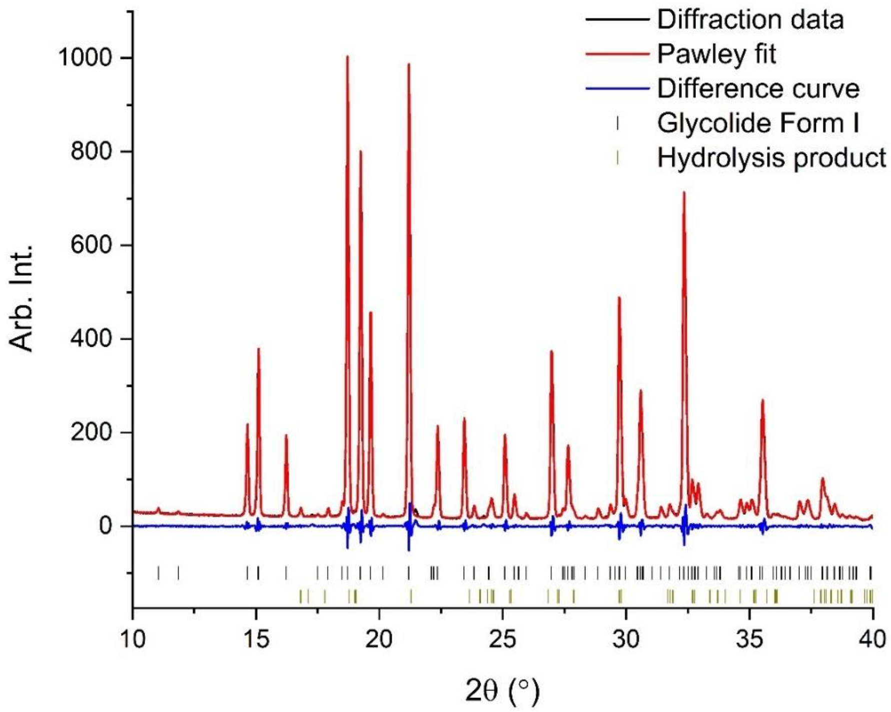

Figure S2 Pawley fit of the Cryomilled sample using glycolide form I as a reference (black tick marks) and the hydrolysis product (2-(2-hydroxyacetoxy)acetic acid; HAA) as a reference (green tick marks).

S1.4 Tablet formulation

S1.4.1 Tablet Preparation

Before blending, micronized glycolide was passed through a stainless steel sieve with a 180 µm apperture to remove any large or agglomerated particles. The sieved glycolide (5 g) was mixed with MCC (19.75 g) by manual geometric blending. Both powders were further mixed using a Pharmatech MultiBlend® MB015 bench top powder blender to ensure powder blend homogeneity, using the following parameters: set blend speed of 20 rpm, set agitator speed of 100 rpm, set blend time of 25 min., set agitator time of 24 min. and delay for agitator of 30 sec. As a final step, MgSt (0.25 g) was sprinkled over the mixture, which was manually tumbled in perpendicular directions 10 times to prevent overmixing and segregation of MgSt. The final powder blend contained glycolide, MCC and MgSt in a 20:79:1 %w/w ratio. During tablet preparation, tablets of the same thickness were compressed at different pressures by applying a 2.15 mm punch separation during compaction and varying the weight of the tablets. The manual method of die fill resulted in a variance in applied pressures defined in the “Tablet name” column in Table S1.

Table S1: Details of the tablets prepared for this study. 20 tablets were selected to be examined using XRD-CT.

Tablet name	Tablet weight (mg)	Actual compaction pressure (MPa)	Was the tablet selected for study?
50_1	52.7	53	-
50_2	52.7	51	-
50_3	52.6	50	Yes
50_4	53.7	55	-
50_5	51.8	47	-
50_6	52.9	52	-

Table S2 (contd.): Details of the tablets prepared for this study. 20 tablets were selected to be examined using XRD-CT.

Tablet name	Tablet weight (mg)	Actual compaction pressure (MPa)	Was the tablet selected for study?
100_1	59.7	92	-
100_2	61.6	104	Yes
100_3	61.5	105	-
100_4	61.2	101	-
100_5	60.9	104	-
100_6	61.4	106	-
130_1	62.2	113	-
130_2	63.9	129	-
130_3	64.9	137	Yes
130_4	64.4	137	-
130_5	64.2	135	-
130_6	64.1	135	-
150_1	65.0	167	-
150_2	62.9	121	-
150_3	64.8	146	-
150_4	64.4	137	-
150_5	65.2	149	-
150_6	65.2	146	-
150_7	65.9	157	-
150_8	66.3	164	-
150_9	65.8	149	-
170_1	63.2	144	-
170_2	65.0	170	-
170_3	64.7	164	-
170_4	64.0	153	Yes
170_5	66.8	198	-
170_6	64.7	166	Yes

Table S3 (contd.): Details of the tablets prepared for this study. 20 tablets were selected to be examined using XRD-CT.

Tablet name	Tablet weight (mg)	Actual compaction pressure (MPa)	Was the tablet selected for study?
200_1	66.1	195	-
200_2	65.8	189	-
200_3	66.6	200	Yes
200_4	67.4	220	Yes
200_5	66.9	211	Yes
200_6	66.7	203	Yes
300_1	70.7	317	Yes
300_2	69.8	296	-
300_3	70.9	329	Yes
300_4	69.6	283	Yes
300_5	71.4	352	Yes
300_6	70.2	299	Yes
400_1	71.8	364	Yes
400_2	75.5	548	Yes
400_3	73.5	440	Yes
400_4	75.1	520	Yes
400_5	73.8	454	-
400_6	73.1	423	-
400_7	72.6	307	Yes
400_8	75.1	392	Yes

S1.4.2 Tablet storage

A DS1923 Hygrochron iButton Temperature/Humidity logger (Measurement Systems Ltd, Berkshire, UK) was used to monitor temperature and humidity during storage. It was stored inside a sealed petri dish, mimicking a sealed container, and recorded the temperature (±0.5°C) and humidity (±5%RH) every 600 seconds for approximately 3 days.

S1.5 X-ray Diffraction Computed Tomography

S1.5.1 Fresh samples

XRD-CT measurements on freshly prepared tablets were performed using EH2 of beamline P07 at the PETRA III synchrotron at DESY. A monochromatic X-ray beam of 74 keV (0.168 Å) was focused to have a spot size of 10 × 1 μm (Horizontal x Vertical). 2D powder diffraction patterns were collected at 100Hz using a Pilatus3 X CdTe 2M hybrid photon counting area detector. The tablets were mounted edge-on onto a goniometer (such that the scan would pass through the tablet top-bottom) which was placed on a rotation stage which was mounted perpendicularly to a hexapod; the hexapod was used to translate the sample across the beam. The XRD-CT scans were measured by performing a series of zigzag line scans in the z (vertical) direction using the hexapod and rotation steps. 600 translation steps and 600 rotation steps over 0-360° range were used per sample with a translation step size of 10 μm and 10ms collection time per point.

S1.5.2 Aged samples

XRD-CT measurements on the same set of tablets aged for 1 month were performed on beamline ID31 of ESRF. Here, the monochromatic X-ray beam energy was 80 keV, focused to a spot size of 20 x 3 μm, and the data was collected at 125 Hz using a Pilatus3 X CdTe 2M hybrid photon counting area detector. 600 translation steps of 10 μm, 10ms collection time per point and 600 rotation steps over 0-360° range were used per sample.

S1.5.3 Data collection and analysis

For both sets of beamtime data the detector calibration was performed using a CeO_2 standard, with each 2D diffraction image being calibrated and azimuthally integrated to a 1D powder diffraction pattern, both without, and with a 10% trimmed mean filter using the pyFAI software package and in-house developed scripts[1,2]

The integrated diffraction patterns were reshaped into sinograms and centered. The XRD-CT images (i.e., reconstructed data volume) were reconstructed using the filtered back projection algorithm. The voxel size in the reconstructed images corresponds to 10 × 10 × 1 μm^3 for the data collected on P07 at DESY and 10 x 10 x 3 μm^3 for data collected on ID31 at the ESRF. To aid interpretation of the data the scattering axis was rescaled to that of a standard lab diffractometer using a Cu Kα source (8.047 keV, 1.5406 Å). Phase identification was performed using Mercury[3] and X'pert Highscore Plus. The simulated data for the identification of phases was taken from: X'pert database (Magnesium stearate, 00-005-0292), Cambridge Structural Database entries (glycolide I and II; NAHNIT01, NAHNIT02; cellulose 1α and 1β; JINROO05, JINROO01).[4,5] Maps of each phase were generated using unique reflections, and composite images created using ImageJ.[6]

S1.5.4 Filtering Process

First to note is the unfiltered data become "streakier" (lines) at higher pressure points indicating the increase in the number of single particles (single crystals) in the beam. This increase in particle size is attributed to the annealing of the crystals under compression. Glycolide is a van der Waals solid, and solids of this type have been known to anneal under pressure, for example, benzene solidifies under high pressure but on solidification it anneals into a single crystal if left.[7] The evidence from these experiments suggests that this is the case here. This causes issues in the reconstruction and prevents our ability to spatially resolve the contents of the tablet. By applying the mean-trimmed approach at 5% and 10% we observe a suppression of the "streaky" artefacts and better resolution and spatial interpretation of the tablet. It should be stressed that whilst we are omitting data, these data reside on the azimuthal rings at the same 2-theta values as the "good" powder rings that have an even distribution of intensity around the ring. Using this staged approach we have been able to understand the annealing process of the crystals of glycolide as higher pressure is applied to the formulation.

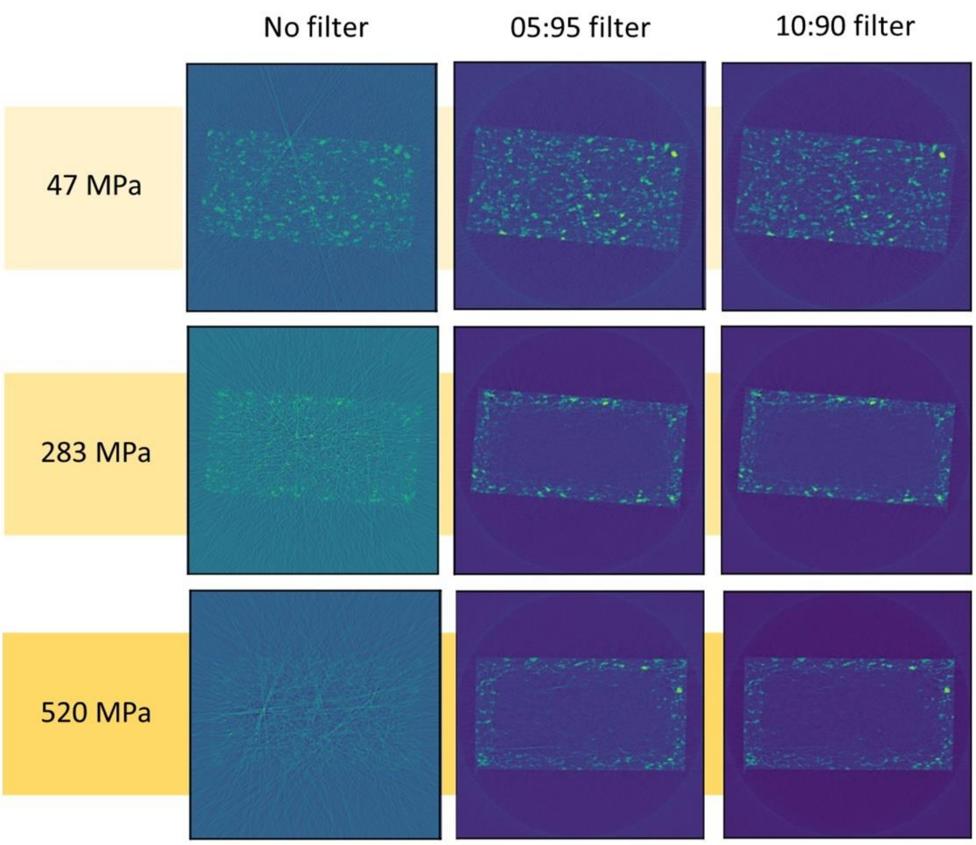

Figure S3: The images represent the various stages of mean-trimmed filtering at different levels using tablets at different compaction pressures. The data represented here is using the 2-theta values of Form I of glycolide (CSD refcode: NAHNIT01[4]).

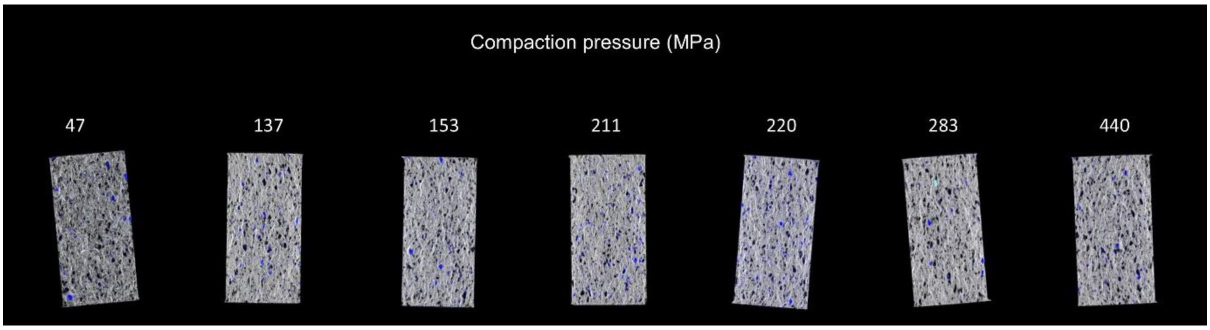

Figure S4: XRD-CT images of tablets compacted at different pressures. MCC is depicted as white and the unknown phases (2-(2-hydroxyacetoxy)acetic acid) is blue.

S1.6 Interpretation of the unknown phase

The global pattern from the aged tablet compressed to 440MPa. This was deconvoluted into the component parts (Figure S5). From this pattern, the unknown phase is the largest component in combination with the broader underlying MCC phase. There is a smaller contribution from glycolide Form II but there is a further phase that was unidentified. Key Bragg diffraction peaks between 16.55 and 16.95 were not associated with the known phases. Due to the larger proportion this phase in the aged tablets our first thought was that the MMC has recrystallized into Cellulose 1β due to the proximity of the peaks at 14.86° and also 16.66°. The recrystallization of MCC is known, however this would be highly unusual as MCC is used extensively as a bulking agent.

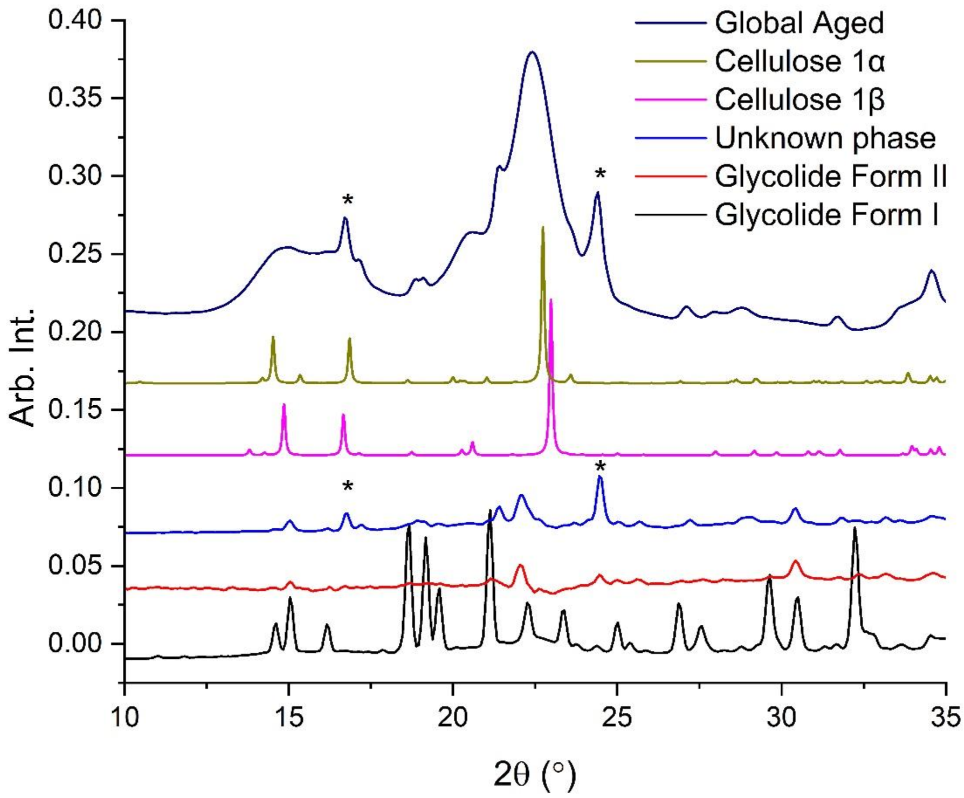

Figure S5: Similarities between peak positions in the X-ray diffraction patterns of glycolide Form I, Cellulose Iα, Cellulose Iß and the unknown phase.

Subsequent experiments on pure glycolide and the blended mixture revealed that the transformation also occurred in these non-tableted samples. Loose powder samples of micronized pure phase glycolide and a blended mixture of glycolide, MCC and MgSt in a 20:79:1 %w/w ratio were prepared following the methods described in Sections S1.2 and S1.4.1. The samples were tested 1, 7, 14, 21, 28, 35, 42, 47, 56, and 302 days after being prepared, using powder X-ray diffraction and following the method described in Section S1.3. The 40 position well plate was lined with two 7.5 µm Kapton films, one above and one below the samples, in order to seal the samples within the plate and minimize their interaction with atmospheric water. We observed that blending the samples increases the rate at which the transformation occurs (Figure S6 & Figure S7). The transformation was monitored over 2 months with a last data point approx. 10 months later. The percentage conversion based on the integration of selected Bragg peaks that are representative of each phase is summarized in Figure S7 (glycolide (002) at 18.68° & (012) at 19.62°, HAA [100] at 16.77° & [011] at 17.22°). The rate at which the transformation occurs in the blend can be attributed to the presence of the MCC. MCC and glycolide are both hygroscopic and the evidence suggests the increased hydration facilitates the conversion. One of the more intriguing elements is that there is transformation of glycolide in its pure state. Storage of unmanipulated glycolide does not appear to transform (Figure S1; purple and blue patterns) so it suggests that the micronisation process of glycolide initiates this to occur. We cryomilled the glycolide to reduce the particle size for the analysis using XRD-CT hence there was potential that some condensation will have occurred in the vessel that might promote the reaction. There is evidence of the HAA solid in the micronized product at a low level (Figure S2).

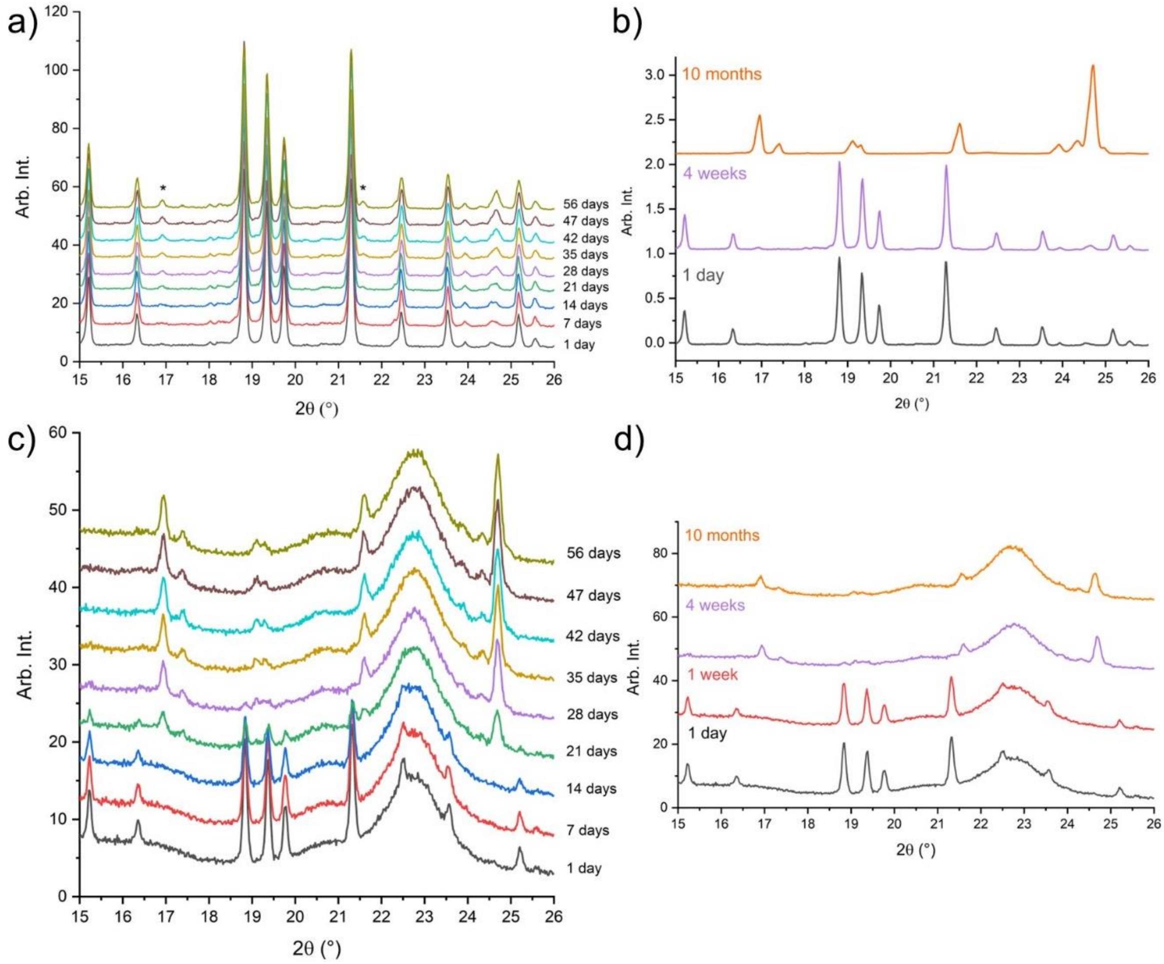

Figure S6: Diffraction patterns for pure glycolide (a & b) and the blended mixture used in the tableting part of the study (c & d). After 21 days there is significant transformation in the blended mixture with complete change in 35 days. This contrasts with the pure material where the transformation begins after 14 days but remains incomplete over 47 days. After approx. 10 months the transformation is complete in the pure substance.

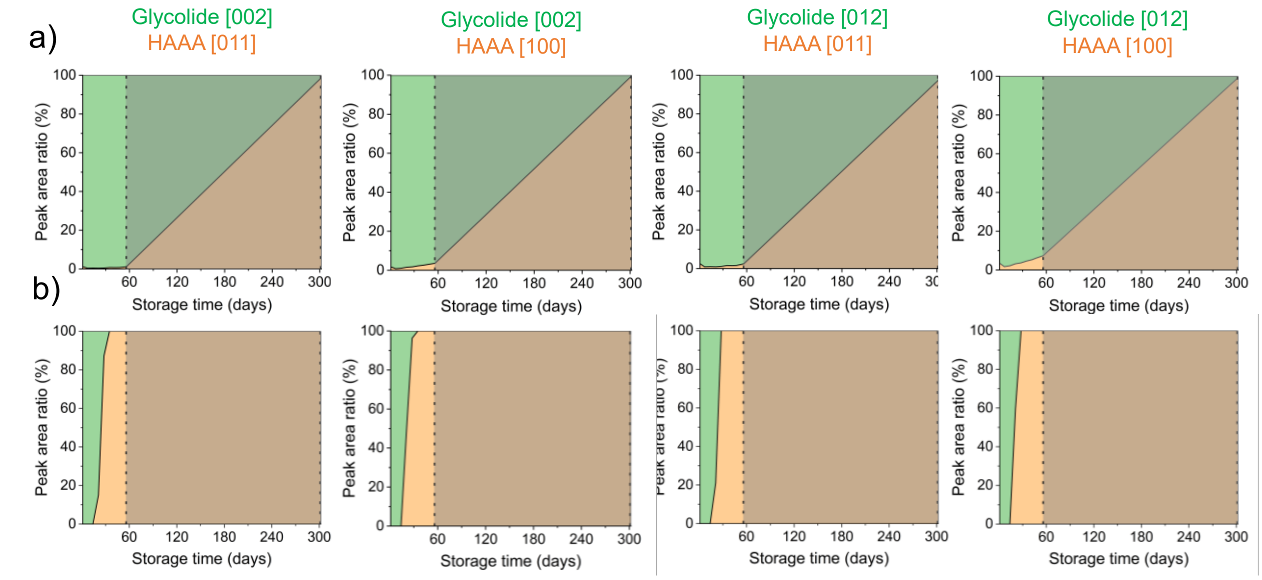

Figure S7: Conversion of glycolide Form I to 2-(2-hydroxyacetoxy)acetic acid in pure (a) and blended (b) mixtures upon storage. The change in the area under two characteristic peaks from the XRPD patterns of each compound is monitored over time. There is a faster conversion observed in the blended mixture over the pure compound. Glycolide peak areas are shown in green, 2-(2-hydroxyacetoxy)acetic acid peak areas are shown in orange. The shadowed area indicates that no data was collected between 2 and 10 months (i.e. shadowed area).

S1.6.1 Electron diffraction

The sample of 2-(2-hydroxyacetoxy)acetic acid was finely dispersed on a standard TEM grid (amorphous carbon on Cu) without grinding and measured on an ELDICO *ED-1* electron diffractometer at room temperature using the software ELDIX.[8] The device is equipped with a LaB_6 source operating at an acceleration voltage of 160 kV (λ = 0.02851 Å) and a hybrid-pixel detector (Dectris QUADRO). Suitable crystals were identified in STEM (scanning transmission electron microscopy) imaging mode and diffraction was recorded in continuous rotation mode with a beam diameter of ca. 750 nm. Parts of measurements showing significant beam damage or shadowing by the grid were omitted. Further data collection details are given in (Table S4).

Table S4: ED data collection details for the crystals of 2-(2-hydroxyacetoxy)acetic acid used in refinement.

**Crystal no.**	**Angular range [°]**	**Rotation per frame [°]**	**Exposure time [s]**	**Total exposure [s]**	**Frames measured**	**Frames used**
1	−60 to +60	1	0.5	120	120	1 to 120
2	−60 to +60	1	0.5	120	120	1 to 55
3	−60 to +60	1	0.5	120	120	1 to 120
4	−60 to +60	1	0.5	120	120	11 to 110
5	−65 to +65	1	0.5	130	130	1 to 50
6	−55 to +55	1	0.5	110	110	1 to 50

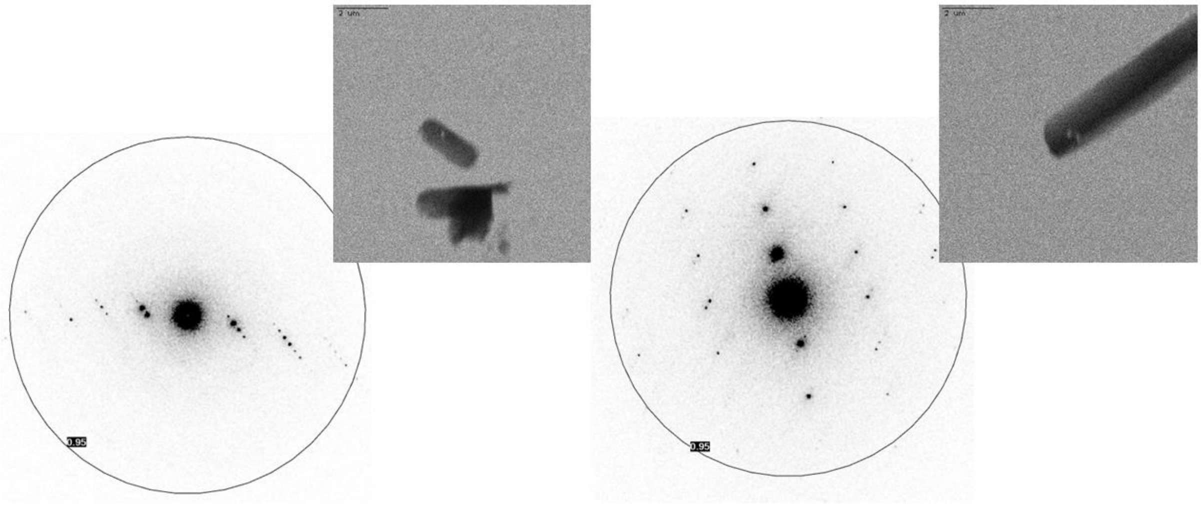

Figure S8: Exemplary diffraction and STEM (field of view 10 µm) images for crystal 1 (left) and crystal 3 (right).

Data were processed and evaluated using the APEX4 software package.[9] After unit cell determination the frames were integrated separately for each crystal, then merged, scaled, and corrected for Lorentz effects, scan speed, background, and absorption using SAINT and SADABS.[10,11] Space group determination was based on systematic absences, E statistics, and successful refinement of the structure. The structure was solved using ShelXT and refined with ShelXL in conjunction with ShelXle.[12–14] Least squares refinements were carried out within the kinematic approximation by minimizing $\Sigma w(F_{obs}^2 - F_{calc}^2)^2$ with the ShelXL weighting scheme and using neutral electron scattering factors.[13,15] H atoms bound to C were placed in calculated positions based on typical distances for neutron diffraction and refined with a riding model and $U_{iso}(H) = 1.2 \cdot U_{eq}(C)$. H atom positions on heteroatoms were refined freely. Deposition Number 2364365 contains the supplementary crystallographic data for this paper. These data are provided free of charge by the joint Cambridge Crystallographic Data Centre and Fachinformationszentrum Karlsruhe Access Structures service and can be accessed at www.ccdc.cam.ac.uk/structures.

S1.6.2 Crystal structure description

2-(2-hydroxyacetoxy)acetic acid crystallises with one molecule in the asymmetric unit in monoclinic $P2_1/c$. The molecules form interactions primarily through the carboxylic acid $R_2^2(8)$ dimer (O1…O2; 2.696(19) Å). At the other end of the molecule the hydroxyl group (O5) is involved in a bifurcated hydrogen bond to O4 and O5 of a neighbouring molecule (2.90(2) Å). The combination of the interactions provides a three-dimensional hydrogen bonded network (Figure S9). The model has been fit to the bulk powder and is well-determined (Figure S10)

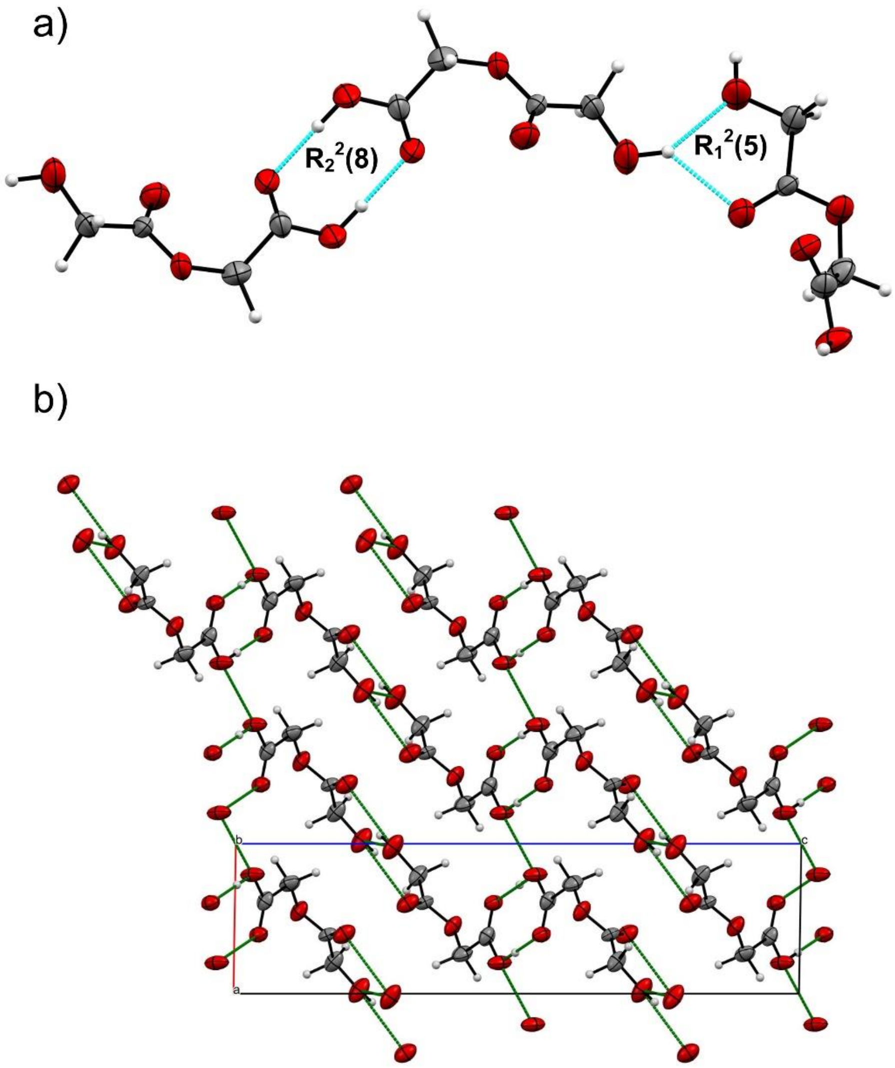

Figure S9: a) The hydrogen bonding of 2-(2-hydroxyacetoxy)acetic acid indicating the carboxylic dimer $R_2^2(8)$ and the bifurcated H-bond between the hydroxyl group and the carbonyl groups ($R_1^2(5)$); b) the packing of the molecules showing the three-dimensional bonding of the structure.

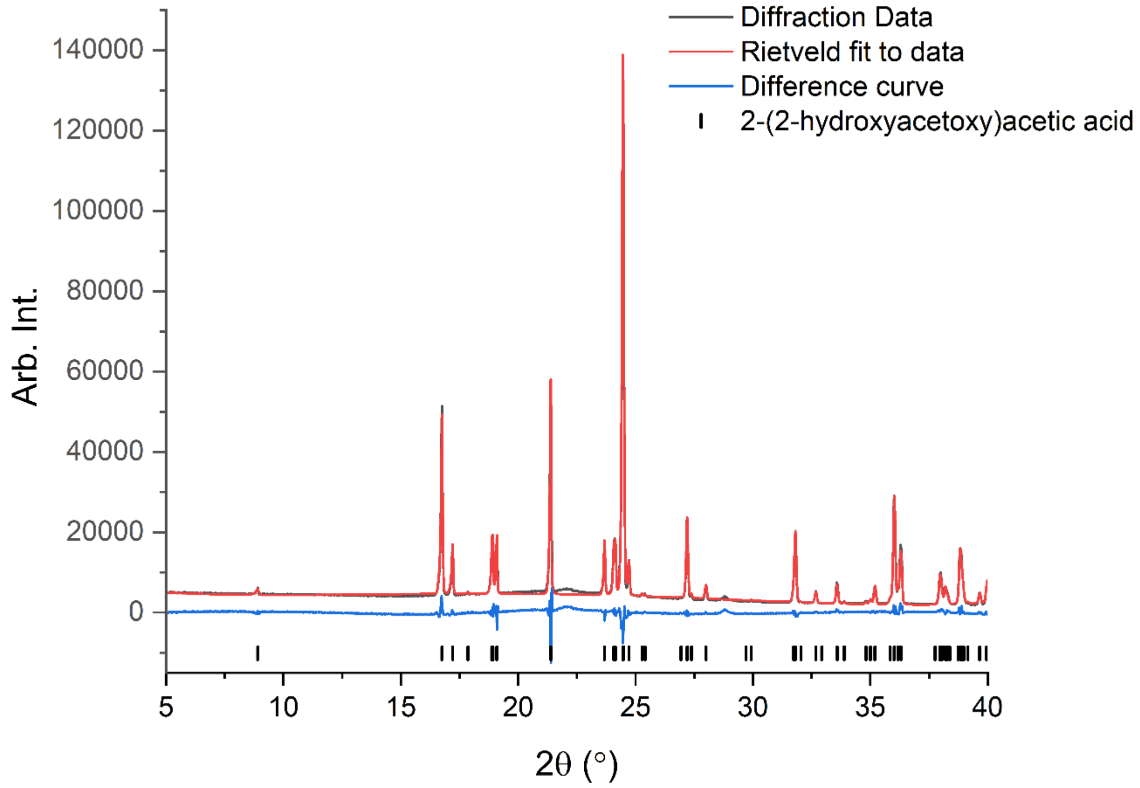

Figure S10: Rietveld fit of 2-(2-hydroxyacetoxy)acetic acid using the coordinates from the crystal structure determination from electron diffraction.

Table S5: The crystallographic parameters for 2-(2-hydroxyacetoxy)acetic acid as determined by electron diffraction and Rietveld analysis of the powder.

	2-(2-hydroxyacetoxy)acetic acid	2-(2-hydroxyacetoxy)acetic acid
Crystal data – Electron diffraction		Crystal data – Rietveld fit
Chemical formula	$C_4H_6O_5$	$C_4H_6O_5$
M_r	134.09	134.09
Crystal system, space group	Monoclinic, $P2_1/c$	Monoclinic, $P2_1/c$
Temperature (K)	298	298
a, *b*, *c* (Å)	5.28 (5), 5.35 (5), 19.92 (19)	5.28703 (18), 5.33231 (16), 19.8459 (7)
β (°)	90.900(13)	90.612(2)
V (Å^3)	562 (9)	559.46(3)
Z	4	4
Radiation type	λ = 0.02851 Å (electrons)	λ = 1.54043 Å (X-rays)
μ (mm^{-1})	0.000	1.349388
Crystal size (mm)	××	Powder
Data collection		
Diffractometer	ELDICO *ED-1*	
Absorption correction	Multi-scan *SADABS2016*/2 (Bruker, 2016/2) was used for absorption correction. wR_2(int) was 0.1704 before	

	and 0.1232 after correction. The Ratio of minimum to maximum transmission is 0.2545. The l/2 correction factor is not present.	
T_{min}, T_{max}	0.189, 0.743	
No. of measured, independent and observed [$I > 2s(I)$] reflections	4316, 847, 693	
R_{int}	0.099	
(sin q/l)$_{max}$ (Å^{-1})	0.602	
Refinement		
$R[F^2 > 2s(F^2)]$, $wR(F^2)$, S	0.159, 0.422, 1.73	R_p = 0.062, R_{wp} = 0.080, R_{exp} = 0.015, χ^2 = 28.933
No. of reflections	847	
No. of parameters	89	
H-atom treatment	H atoms treated by a mixture of independent and constrained refinement	
Δρ$_{max}$, Δρ$_{min}$ (e Å^{-3})	0.18, -0.22	

Computer programs: Eldix[8], *SAINT*[10], SHELXT[12], SHEXL,[13] Olex2 1.5[16]

References:

[1] A. Vamvakeros, S. D. M. Jacques, M. Di Michiel, V. Middelkoop, C. K. Egan, R. J. Cernik, A. M. Beale, *J. Appl. Crystallogr.* **2015**, *48*, 1943–1955.

[2] G. Ashiotis, A. Deschildre, Z. Nawaz, J. P. Wright, D. Karkoulis, F. E. Picca, J. Kieffer, *J. Appl. Crystallogr.* **2015**, *48*, 510–519.

[3] C. F. Macrae, I. Sovago, S. J. Cottrell, P. T. A. Galek, P. McCabe, E. Pidcock, M. Platings, G. P. Shields, J. S. Stevens, M. Towler, P. A. Wood, *J. Appl. Crystallogr.* **2020**, *53*, 226–235.

[4] C. R. Groom, I. J. Bruno, M. P. Lightfoot, S. C. Ward, *Acta Crystallogr. Sect. B Struct. Sci. Cryst. Eng. Mater.* **2016**, *B72*, 171–179.

[5] I. B. Hutchison, A. Delori, X. Wang, K. V. Kamenev, A. J. Urquhart, I. D. H. Oswald, *CrystEngComm* **2015**, *17*, 1778–1782.

[6] C. A. Schneider, W. S. Rasband, K. W. Eliceiri, *Nat. Methods* **2012**, *9*, 671–675.

[7] L. Ciabini, F. A. Gorelli, M. Santoro, R. Bini, V. Schettino, M. Mezouar, *Phys. Rev. B* **2005**, *72*, 094108.

[7] ELDIX Software Suite, Eldico, **2023**.

[8] APEX 4, Bruker AXS Inc, **2022**.

[9] SAINT, Bruker AXS Inc, **2019**.

[11] G. M. Sheldrick, **2016**.

[12] G. M. Sheldrick, *Acta Crystallogr. Sect. Found. Adv.* **2015**, *71*, 3–8.

[13] G. M. Sheldrick, *Acta Crystallogr. Sect. C Struct. Chem.* **2015**, *71*, 3–8.

[14] C. B. Hübschle, G. M. Sheldrick, B. Dittrich, *J. Appl. Crystallogr.* **2011**, *44*, 1281–1284.

[15] L.-M. Peng, *Acta Crystallogr. A* **1998**, *54*, 481–485.

[16] O. V. Dolomanov, L. J. Bourhis, R. J. Gildea, J. A. K. Howard, H. Puschmann, *J. Appl. Crystallogr.* **2009**, *42*, 339–341.
